# Clinical Remarks on Erysipelas

**Published:** 1871-11

**Authors:** Thos. F. Rochester

**Affiliations:** The Hospital of the Sisters of Charity


BUFFALO

### Medical and Surgical Journal.

VOL. XI. NOVEMBER, 1871. No.4.

### Original Communications.

:o:

*Clinical Remarks on Erysipelas*, by PROF. THOS. F. ROCHESTER, in the Hospital of the Sisters of Charity.

Reported by Edward N. Brush, Member of the Class.

GENTLEMEN:—

Your attention has been called, while passing through the Wards, to a case of Erysipelas.

This is an affection, inflammatory in character, of peculiar and asthenic type. Erysipelas, like Diphtheria, is a general disorder, being locally confined, but indicating a certain degree of disease in the whole system. It is a term which is often used with impropriety, being applied to Erythmea, Acne, chronic red face, nose, or an eruption of any kind. It develops itself sometimes in newly-born children, generally at the umbilicus; a slight irritation from traction on cord, or want of cleanliness on the part of the nurse is often the cause, but it is also often a puerperal condition of maternal origin. Erysipelas manifests itself at all ages, but proves the most fatal in the extremes of life. Generally speaking, it is an inflammation of the derma or true skin. Blister or vesication is more apt to occur where the integument is stretched over a hard substance, as the cartilage of the ear or bones of the nose. The inflammation sometimes extends to the deep structures involving the sheaths, but not the muscles themselves.

There is a form of Erysipelas which is hinted at in Gross' Surgery, a very peculiar and a very fatal form. It generally commences at the angle of the eye, and is not accompanied by any constitutional trouble. A specific peculiarity is that you get an Erysipelatous inflammation of the mucous membrane of the eye. The eye lids become inflamed and swollen, and in a short time the eyesight is entirely shut off by desquamative conjunctivitis, the membrane rolling up into scrolls.

I have seen four cases of this character, and I have seen four fatal results. The manner of death would indicate that the brain is involved. Perhaps the disease extends itself through the optic structures to the base of the brain. The cases which I saw were in persons between twenty and forty years of age.

In the first case I had no idea of danger. General hygienic measures were adopted. In a short time the patient died. The second patient died the second day after I was called in consultation. The death of the third I attributed more to complication with other diseases than to a severe attack of Erysipelas. The fourth called at my office, he was a captain on the lakes, had no bad habits, was not addicted to drink, was not syphilitic. I noticed the characteristic inflammation of the eye, and told him to go home and to bed. His pulse never varied from about 70 per minute. There was no vomiting, no diarrhœa; in about ten days his eyes were well, and I put him down as one successful case. One Saturday I told him I should not call to see him the next day as he was so much improved, but being in the vicinity I ran in to see him. His wife met me, saying that her husband had been up for a short time, but had gone back to bed again. I went into his bedroom and found him dying. I think effusion had taken place about base of the brain.

Erysipelas is one of those diseases called zymotic, it is a blood disease. It sometimes, as an epidemic, gives rise to that disease called black tongue. It prevails chiefly in that season of the year which is cold and damp. Not only does it extend by atmospheric influence, but may be carried by one person to another. It is never safe to make a surgical operation after visiting a patient who has Erysipelas. When Erysipelas is prevalent, Scarlet Fever almost always is, and *vice versa*. It may also give rise to Puerperal Fever. It is dangerous for a woman about to be confined when Erysipelas is prevalent. A medical man should always take great care in attending cases of parturition and Erysipelas, lest by some means the parturient female suffer from puerperal fever, the result of contagion.

The treatment of Erysipelas should be both local and general. Many practitioners think topical applications of little value, and some regard them as even hurtful. Others, on the contrary, depend almost exclusively upon them. I suspect that in this, as in most matters of controversy, "the truth lies between." Topical measures should have for their object, the arrest of the progress of the disorder and the alleviation of the pain and itching. The tincture of the chloride of iron undiluted, applied with a hair pencil, has almost superceded the tincture of Iodine. Velpeau's lotion is a strong solution of the sulphate of iron in water, and is admirably adapted to large surfaces; housekeepers, however, will complain of its effect upon the sheets and pillow cases. Twenty years ago the "house lotion" at Bellevue was the following :

| | | |
|---|---|---|
| ℞ | Plumbi Acetatis, - - - - - | ʒj. |
| | Aquæ Puræ, - - - - - | ℥xxxij. |
| | Tr. Opii, - - - - - | ℥ij. |

and it is still the most popular application there.

Whisky, pure or diluted, has its advocates, simple warm water is also recommended, and the dry powder of starch or flour is much used. Attempts are sometimes made to establish a line of demarkation with vesicants, or with the solid nitrate of silver. The popular cranberry poultice owes its efficacy to the acid juice of the fruit.

The general treatment of Erysipelas should, from the nature of the disorder, be sustaining and expectant. Often little or no medication is required, and nearly as often therapeutic agents are demanded, and there is room for much judgment and skill in their selection and adaptation to individual cases. Purgation as remedial is not to be advised, except where coma occurs, and then must be employed cautiously. Diuretics and dilutents, in the shape of large draughts of pure water, or with a slight alkaline admixture, are not objectionable, and when the urine is scanty, bloody, or albuminous, should always be used. Restlessness and wakefulness require anodynes. Dovers Powder with Quinine often answer well, but failed in the patient you have seen to-day. Last night he took twenty grains of Chloral hydrate, slept ten hours, and awoke refreshed, and with his mind clear. I imagine it will often act just in this happy way—but I have never prescribed it before in this affection. There are very few cases of Erysipelas that do not get on well with Quinine, or with Quinine and the mineral acids. The tincture of the chloride of Iron is, if possible, more in demand for internal than external use. It was first advocated on the hypothesis, that the disease was associated with a great deficiency of the red corpuscles of the blood, and that iron in solution was to be used to correct this condition. Whether the hypothesis is just or not, the remedy proposed met with a very favorable reception, and has long been employed, and appears to be in as great favor as ever. In Erysipelas and in all kindred asthenic disorders, I think you will find the following combination useful. I have used it so often, and so successfully, that I am very partial to it.

℞ Potass Chlor. Pul. - - - - ℥ss.

Tr. Ferri. Chlor. - - - - - ℥j.

Syr. Simplicis, - - - - - ℥v.

Quiniæ Sulph. - - - - - ʒjss.

M.

Make a solution.

A teaspoonful in water every two to four hours.

Phlegmonous Erysipelas, in addition to the local and general treatment above described, usually requires deep and free incisions. Dilute solutions of carbolic acid and of permanganate of potash, are advised locally, conjointly with the internal administration of the sulphite of soda.

